# Advances in endothelial cell targeting by AAV vectors

**DOI:** 10.3389/abp.2026.15740

**Published:** 2026-02-09

**Authors:** Milena Cichon, Alicja Jozkowicz, Anna Grochot-Przeczek

**Affiliations:** 1 Department of Medical Biotechnology, Faculty of Biochemistry, Biophysics and Biotechnology, Jagiellonian University, Kraków, Poland; 2 Doctoral School of Exact and Natural Sciences, Jagiellonian University, Kraków, Poland

**Keywords:** AAV, adeno-associated virus, blood vessels, endothelial cells, gene therapy

## Abstract

Adeno-associated virus (AAV) vectors have become a cornerstone of *in vivo* gene delivery. However, although the endothelium is the first cellular interface encountered after systemic delivery, native AAV serotypes exhibit poor endothelial transduction, favoring hepatocytes, muscle cells and, neurons instead. This limitation represents a major barrier to gene therapies targeting cardiovascular, neurovascular, and inflammatory diseases. This review summarizes recent advances in redirecting AAV tropism toward endothelial cells (ECs) through genetic capsid engineering, peptide display, and non-genetic surface modification. We highlight the previously underrecognized endothelial tropism of the AAV4 serotype, attributed to its unique recognition of O-linked sialic acids. We also describe multiple approaches to capsid retargeting, including the incorporation of EC-binding peptides that enable cell entry into specific vascular beds, as well as genetic engineering strategies that reduce heparan sulfate proteoglycan (HSPG) binding and hepatocyte transduction while enhancing intracellular trafficking in ECs. In addition, we discuss polymer-coating approaches that allow receptor-specific targeting of ECs with reduced recognition by immune cells. Together, these strategies represent promising avenues for enhancing vascular tropism and transduction efficiency of modified AAVs, moving the field closer to precise vascular gene therapies.

## Introduction

Adeno-associated viruses (AAVs) have emerged as one of the most promising viral vectors in gene therapy due to their favorable safety profile and ability to mediate gene expression in both dividing and non-dividing cells. AAVs are non-cytopathic, elicit relatively mild immunogenicity compared with other viral vectors, and only rarely integrate into the host genome (Zhang et al., 2022).

**FIGURE 1:**
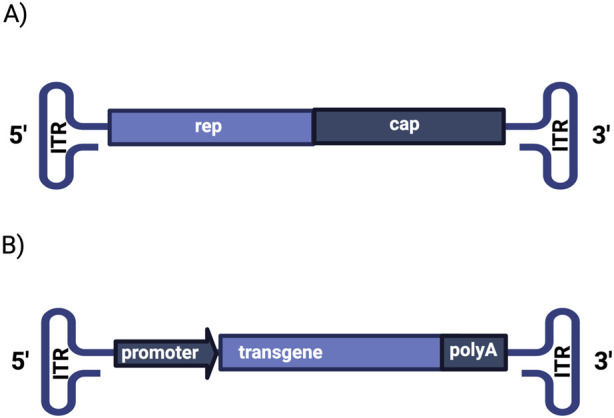
Schematic representation of **(A)** wild-type and **(B)** recombinant AAV genome structure. The genome of wild-type AAV contains the *rep* (replication) and *cap* (capsid) genes flanked by inverted terminal repeats (ITRs). Recombinant AAV vectors are constructed by replacing *rep* and *cap* with a transgene of interest under the control of specific promoter and polyadenylation signal.

Wild type AAV particles are small (∼25 nm), non-enveloped viruses belonging to the family *Parvoviridae*, genus *Dependovirus* (Berns and Giraud, 1996; Pajusola et al., 2002). Their genome is a single-stranded linear DNA of approximately 4.7 kilobases in length. It consists of two open reading frames (ORFs), *rep* and *cap*, flanked by two 145-base-pair inverted terminal repeats (ITRs) that are essential for genome replication and packaging. The rep gene encodes proteins required for genome replication and site-specific integration, while the cap gene encodes the structural capsid proteins VP1, VP2 and VP3. These three proteins are expressed at a stoichiometry of 1:1:10, respectively, and are assembled into the subunits of the icosahedral AAV particle (Suarez-Amaran et al., 2025). In recombinant AAVs (rAAVs), *rep* and *cap* can be replaced with a therapeutic transgene expression cassette, which typically includes the gene of interest under the control of a specific promoter, and regulatory elements for cell-specific expression (Chamberlain et al., 2017) (Figure 1).

Wild-type AAV has the unique ability to integrate site-specifically into the AAVS1 locus located within the first intron of the *Ppp1r12c* (protein phosphatase 1 regulatory subunit 12C) gene on human chromosome 19, a process mediated by the viral Rep protein. In rAAV vectors, however, the *rep* and *cap* genes are replaced by the transgene expression cassette, flanked only by the ITRs (Nicklin and Baker, 2002; Chamberlain et al., 2017; Lyu et al., 2018). This modification eliminates site-specific integration. As a result, rAAV vectors generally persist episomally in the nucleus, with integration events rare and occurring randomly rather than at AAVS1 (Schultz and Chamberlain, 2008). Such episomal persistence, combined with the low genotoxic risk, significantly improves the safety profile of rAAV vectors (Lyu et al., 2018). However, under conditions of helper virus coinfection, AAV genomes may undergo robust replication causing severe cellular stress. High vector doses and the presence of pre-existing anti-AAV immunoglobulins can also lead to increased AAV-related pathogenicity and toxicity (Ertl, 2022). Of note, the AAVS1 locus itself has been widely used in genome editing as a “safe harbor” site, since targeted insertions there are generally well tolerated and support stable expression (Lyu et al., 2018).

**FIGURE 2:**
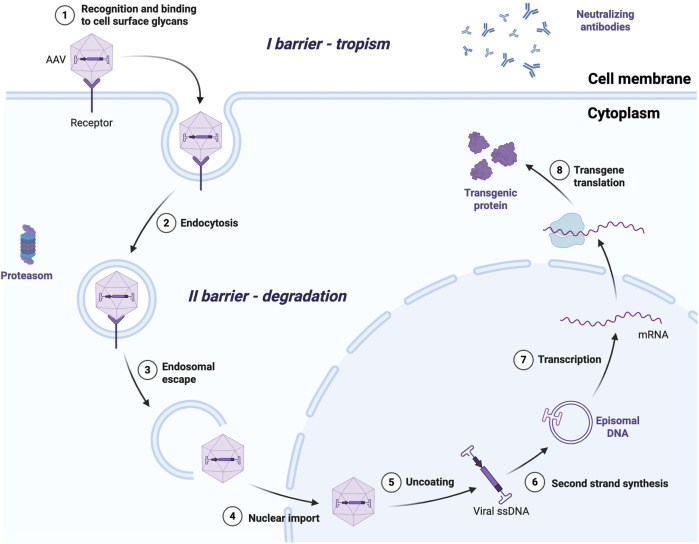
Infection and cellular trafficking of AAV vector. AAV infection steps include recognition of a serotype-specific receptor, internalization via endocytosis, successful endosomal escape, nuclear import, uncoating, transcription from episomes and transgene translation. For the successful transduction of targeted tissues vectors with specific tropism are needed. Once inside the cell AAVs must resist acidification, escape from endosomes and avoid proteasomal degradation. They enter nucleus via nuclear pores, uncoat and convert their single-stranded genome to double-stranded DNA usually in the form of episomes, but in rare cases it can integrate into the host genome. The dsDNA genome serves as a template for transcription of the transgene mRNA and subsequent translation of the encoded protein. (Image was created with BioRender. (2025). AAV Vector Infection. https://app.biorender.com/biorender-templates/details/t-f932759dc9c9200a28fb4be-aav-vector-infection).

Following systemic administration, AAV vectors must traverse multiple biological barriers to achieve transduction. These include evading neutralization by pre-existing host antibodies, binding to cell-surface glycans (which vary by serotype), and internalization via endocytosis. Once inside the cell, they encounter a harsh intracellular environment, where they must escape from endosomes, undergo cytoplasmic trafficking, nuclear import, uncoating, and second-strand synthesis to yield transcription-competent episomes (Figure 2). Each of these steps is influenced by capsid structure and host-cell interactions, ultimately determining tissue tropism and transduction efficiency (Riyad and Weber, 2021; Walkey et al., 2025). One of the rate-limiting steps in rAAV transduction is the conversion of the single-stranded DNA (ssDNA) genome into transcriptionally active double-stranded DNA (dsDNA). In conventional single-stranded vectors (ssAAV) this process relies on host-cell-dependent second-strand synthesis. This limitation can be circumvented through the use of self-complementary vectors (scAAVs), which package genomes that rapidly self-anneal after uncoating, creating dsDNA. scAAVs exploit a natural by-product of the AAV replication cycle – dimeric inverted repeat genomes (McCarty, 2008). Specifically, failure of the Rep endonuclease to nick the terminal resolution site (trs) before the replication fork reaches the downstream ITR, permits replication to proceed through the ITR, generating a covalently linked dimeric genome (Zhang et al., 2024). To favor production of such genomes, scAAV vectors are engineered to lack a functional trs in one of the ITRs, thereby enforcing packaging of a self-complementary genome that efficiently self-anneals after decapsidation and effectively bypasses the second-strand synthesis step (McCarty et al., 2003). This modification dramatically enhances transgene expression, with reported increases ranging from 5- to 140-fold compared to ssAAV vectors *in vitro* (Hacker et al., 2005; Lee et al., 2007). However, this improvement comes at the cost of a substantially reduced packaging capacity of the already small vector, decreasing maximum construct size from approximately 4.7 kb–2.5 kb (McCarty, 2008).

In diseases requiring delivery of large coding sequences, such as hemophilia B, Duchenne muscular dystrophy, cystic fibrosis, or in CRISPR-Cas-based genome-editing approaches, this restricted packaging capacity represents a major limitation (Kolesnik et al., 2024). Several strategies have been developed to address this constraint. One approach involves redesigning large transgenes as minigenes (Wang et al., 2000; Ran et al., 2015; Excoffon et al., 2024), along with the use of minimal functioning copies of ITRs (Savy et al., 2017), promoters (Graßhoff et al., 2022; Wang et al., 2023) and posttranscriptional regulatory elements (Choi et al., 2014). A prominent example of the minigene strategy is Elevidys, an approved gene therapy for Duchenne muscular dystrophy that employs a micro-dystrophin transgene (Hoy, 2023). However, the use of minigenes often results in reduced transduction efficiency and diminished transgene expression (Kolesnik et al., 2024) An alternative strategy relies on splitting of the genes across multiple AAV vectors, most commonly dual- or triple-vector systems. These include trans-splicing approaches, in which splice donor and splice acceptor signals are positioned at the termini of the packaged cDNAs, as well as strategies based on overlapping sequences that enable reconstitution through homologous recombination (Kolesnik et al., 2024). Inteins can also be employed for gene splicing. These non-mammalian, self-splicing protein segments are encoded within transgenes and excised post-translationally, allowing ligation of its flanking protein fragments (exteins) (Shah and Muir, 2014). Notably, inteins may trigger unwanted immune responses due to the presence of foreign epitopes. Multi-vector strategies generally suffer from markedly reduced efficiency. Successful transgene reconstitution requires that multiple vectors simultaneously target the same cell, undergo internalization, traffic to the nucleus, uncoat and accurately reassemble the split gene segments (Kolesnik et al., 2024). This challenge is particularly pronounced in endothelial cells (ECs), where transduction efficiency is already limited, even with the modified AAV capsids discussed in this review.

## Endothelial cells as targets for AAV-based gene delivery

ECs form the inner lining of blood and lymphatic vessels and are central regulators of vascular tone, permeability, leukocyte recruitment, angiogenesis, and hemostasis. They play a pivotal role in the pathogenesis of cardiovascular diseases such as atherosclerosis, thrombosis, pulmonary hypertension, neurovascular disorders, and inflammatory syndromes (Rajendran et al., 2013). Therefore, targeting ECs with viral gene therapies holds significant therapeutic potential.

Because ECs are in direct contact with circulating blood, they are the first cell type encountered by intravenously administered vectors (White et al., 2004). Despite this anatomical advantage, most native AAV serotypes transduce ECs very poorly, instead displaying strong tropism for hepatocytes, neurons, or muscle tissue (Wang et al., 2024). Certain serotypes, such as AAV1 and AAV5, show slightly higher EC transduction than others, but still with much lower efficiency than in their primary target tissues (Chen et al., 2005). The only known natural serotype of AAV with a relatively high endothelial tropism is AAV4, which has recently been reported to efficiently target not only epithelial cells, but also ECs (Walkey et al., 2025). After intravenous injection, AAV vectors preferentially accumulate in the liver or heart rather than the vascular endothelium (Wang et al., 2024). Developing efficient methods for targeting ECs *in vivo* is therefore of considerable therapeutic interest. Endothelium-targeted gene delivery could (1) restore or modulate the expression of enzymes, receptors, or adhesion molecules in systemic vascular diseases; (2) correct genetic disorders affecting vascular homeostasis, such as von Willebrand disease or hemophilia; (3) deliver anti-inflammatory or anti-thrombotic factors directly to sites of pathology; or (4) enable modulation of endothelial function in specific organs without impacting other tissues. Thus, achieving selective and efficient EC transduction is a critical step toward translating vascular-targeted gene therapies into clinical practice (Rajendran et al., 2013).

## Viral vector delivery limitations

The use of viral vectors still faces many limitations. A persistent challenge in clinical translation is the host immune response to AAV capsids. Wild-type AAVs naturally infect humans and other mammals, resulting in pre-existing neutralizing antibodies (nAbs) against one or more AAV serotypes, which limit vector bioavailability and prevent re-administration (Chamberlain et al., 2017; Kaygisiz and Synatschke, 2020). Current approaches to overcome this challenge include re-engineering the AAV capsid, such as mutating conserved residues targeted by nAbs (Bartel et al., 2011; Tse et al., 2017; Lugin et al., 2020), combining different serotypes to yield chimeric vectors (Hauck et al., 2003), or applying directed evolution to large structurally guided libraries of AAV capsids (Pekrun et al., 2019). Other strategies include using empty capsids as decoys (Mingozzi and High, 2013), coating the surface with polyethylene glycol (PEG) (Lee et al., 2005), encapsulating them in hydrogels or liposomes (Pinto et al., 2025), anchoring AAVs to larger vehicles like red blood cells (Zhao et al., 2022), or using immunoglobulin-degrading enzymes (Elmore et al., 2020). These approaches either reduce immune responses–protecting the host from the severe effects of inflammation – or delay AAV neutralization, thereby enabling more successful vector re-administration and increasing transgene expression efficiency (Kaygisiz and Synatschke, 2020). However, the clinical applicability remains limited. These methods may themselves provoke immune or allergic reactions (Pinto et al., 2025). Genetic modification of capsids frequently disrupts proper folding and requires precise combination and number of mutations to preserve infectivity. PEGylation presents additional constrains, as both size and site-specific attachment of PEG chains are critical. Only a narrow molecular weight range effectively reduces immunogenicity, whereas suboptimal PEGylation markedly diminishes vector infectivity. Therefore, PEG-based approaches require highly standardized production protocols and stringent quality-control measures (Yao et al., 2017; Pinto et al., 2025). To date, most immune evasion strategies have shown modest efficacy, with only immunoglobulin G-degrading enzymes approach progressing toward clinical trials (Cao et al., 2025).

Moreover, transduction can be hindered by insufficient viral tropism that limits cell entry, poor trafficking to the endosome, slow endosomal escape, and ineffective nuclear transport, followed by reduced second strand synthesis (Ferrari et al., 1996; Kaygisiz and Synatschke, 2020). One contributing factor to poor EC transduction is the rapid proteasomal degradation of internalized vectors before they can translocate to the nucleus. This barrier can be partially overcome in experimental systems by using proteasome inhibitors such as MG101 (N-acetyl-L-leucyl-L-leucyl-L-Norleucinal; LLnL) or MG132 (carbobenzoxy-leucyl-leucyl-leucinal; Z-LLL), which enhance transgene expression (Nicklin et al., 2001; Pajusola et al., 2002).

## AAV serotypes and endothelial tropism

The tropism of the AAV vector is primarily determined by the structural composition of the viral capsid, which dictates its interaction with specific cell-surface receptors and subsequent internalization. Different AAV serotypes possess distinct capsid proteins, resulting in varying receptor affinities and divergent tissue tropism.

Capsid-host cell interactions are mediated by vector recognition of distinct glycans. For example, AAV1, AAV5, and AAV6 bind to N-linked sialic acids (α2-3 or α2-6 linkages) (Walters et al., 2001; Huang et al., 2016), whereas AAV4 binds to α2-3 O-linked sialic acid (Walkey et al., 2025). AAV2 and AAV3 preferentially bind heparan sulfate proteoglycans (Kern et al., 2003; Perabo et al., 2006; Lerch and Chapman, 2012), and AAV9 recognizes N-linked galactose residues (Bell et al., 2012). A major limitation in the use of conventional AAV serotypes is their strong bias toward liver transduction, largely due to the high expression of AAVR (Adeno-Associated Virus Receptor) in hepatocytes. AAVR is a broadly expressed transmembrane protein that serves as a crucial post-binding co-receptor for many AAVs, facilitating intracellular trafficking steps such as endosomal escape and nuclear entry (Summerford et al., 2016). The hepatic preference of AAVs presents a significant hurdle in efforts to target other organs.

However, receptor binding alone does not fully explain the AAV tissue tropism *in vivo*. Vector distribution and transduction are determined by a complex interplay of factors that include species, sex, age, vector dose, route of administration, circulation kinetics, tissue vascular permeability, receptor expression density, intracellular trafficking pathways and host immune responses (Walkey et al., 2025). For example, AAV1 and AAV5 exhibit high specificity for human aortic endothelial cells (HAECs) and rat aortic endothelial cells (RAECs) *in vitro*, but only moderate transduction *in vivo* in rats (Chen et al., 2005).

Recent studies have revealed a previously underrecognized pan-endothelial tropism of AAV4 in mice, a serotype that has not been extensively used due to its low production titers and limited characterization. In contrast to liver-tropic serotypes, AAV4 displays minimal transduction of hepatocytes and sinusoidal liver ECs in mice. Instead, it shows a strong preference for ECs, particularly in the lung (with up to 80% of lung ECs transduced), as well as in the heart and diaphragm (Walkey et al., 2025). Moreover, AAV4 enables endothelial gene transfer in a wide range of murine peripheral tissues, including the stomach, bladder, adrenal glands, pancreas, brain, small and large intestines, thymus, skeletal muscle, lymph nodes, skin, ovary, uterus, and retina, albeit with lower efficiency than in the lung. Transduction of thoracic aortic endothelium reached up to 26%, highlighting AAV4 potential for systemic vascular applications (Walkey et al., 2025).

The capsid of AAV4 displays a unique affinity for α2-3 O-linked sialic acid, which may underlie its preferential tropism for the cardiopulmonary endothelium. O-linked sialic acids are more abundant and accessible on ECs and the lung epithelium, in contrast to hepatocytes, which predominantly exhibit N-linked glycans (King et al., 2017). Moreover, AAV4 cell entry is AAVR-independent, indicating the reliance on an alternative, yet unidentified entry mechanism. This receptor independence likely contributes to its reduced liver tropism. Although the intracellular mechanisms governing AAV4 endothelial selectivity remain incompletely understood, AAV4 appears to be a promising candidate for endothelial-targeted gene therapy applications, particularly in the pulmonary vasculature and other non-hepatic vascular beds (Walkey et al., 2025). However, results obtained in mice cannot be directly extrapolated to other species, including humans. For example, efficient endothelial transduction by AAV4 could not be confirmed in rats (Chen et al., 2005). Importantly, a recently reported head-to-head comparison of AAV serotypes demonstrated relatively poor performance of AAV4 – lower than AAV1, AAV2, AAV3 and AAV6 – in transduction of human brain microvascular endothelial cells (HBMECs), human cardiac microvascular endothelial cells (HCMECs), human pulmonary microvascular endothelial cells (HPMECs), human renal glomerular endothelial cells (HRGECs), and human hepatic sinusoidal endothelial cells (HHSECs), while showing relatively high transduction of the HepG2 human hepatoblastoma cell line (Stamataki et al., 2025).

## Strategies to overcome endothelial resistance to AAVs

**FIGURE 3:**
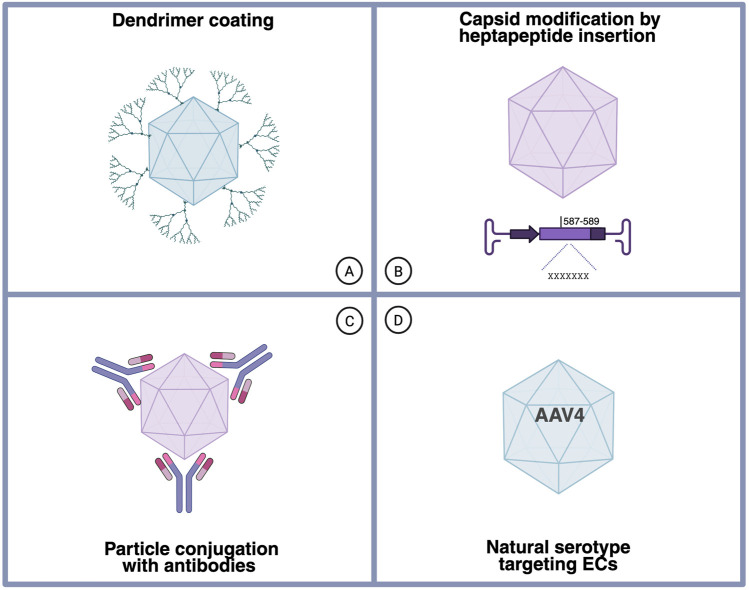
Strategies to enhance endothelial tropism of AAV vectors. **(A)** Surface coating of AAV particles with endothelial-affine polymers. **(B)** Genetic capsid engineering by the insertion of endothelial-targeting peptides into the exposed loop around amino acid position 587–589 of the VP3 protein in AAV genome. **(C)** Chemical conjugation of the viral capsid with antibodies specific for endothelial cell surface markers. **(D)** The use of naturally occurring AAV serotypes with innate endothelial preference, particularly AAV4.

ECs are remarkably resistant to native AAV transduction, which presents a key challenge for vascular-targeted gene therapies. Multiple strategies have been developed to improve AAV-mediated gene delivery to ECs. Early efforts focused on modifying the heparan sulfate proteoglycan (HSPG) binding site of AAV2 (Nicklin et al., 2001). Subsequent approaches included peptide biopanning to identify endothelial-targeting peptides (Nicklin et al., 2001; White et al., 2004; Barbon et al., 2023), such as those targeting the brain vasculature (Chen et al., 2009; 2023; Deverman et al., 2016; Körbelin et al., 2016; Ravindra Kumar et al., 2020; Krolak et al., 2022), replacement of surface-exposed amino acids on capsids to redirect AAV tropism (Nicklin et al., 2001), the polymer coating of AAV particles with dendrimer-peptide conjugates (Bozoglu et al., 2022; Noormalal et al., 2024), and the attachment of monoclonal antibodies against endothelial surface proteins to the viral capsid (Pearce et al., 2019) (Figure 3). Genetic modification of the viral capsid can enhance EC transduction via novel receptors and trafficking pathways. Interestingly, recent studies have also identified unmodified AAV serotypes with inherent endothelial tropism, particularly in the pulmonary vasculature (Walkey et al., 2025).

## Capsid genetic modifications

ECs are highly heterogeneous, and display distinct surface receptor repertoires across tissues and vascular beds. This heterogeneity suggests that precise targeting strategies are required to achieve organ- or site-specific gene delivery to ECs. Therefore, retargeting AAV capsids – removing native tropism and conferring EC-specific recognition – is a critical step in developing systemically injectable vectors for vascular applications. Beyond receptor engagement, capsid modifications can also alter intracellular trafficking of viral particles, including their sensitivity to endosomal pH and susceptibility to degradation pathways (Nicklin and Baker, 2002), as well as viral uncoating in the nucleus (Zhang et al., 2019).

A widely used strategy for retargeting EC involves incorporating short, endothelium-homing peptides, derived from phage display libraries, into specific sites in the viral capsid (Table 1). Most commonly, these peptides are inserted at positions 587–589 of the VP1 protein, a site that has been shown to tolerate peptide insertions without disrupting capsid assembly or genome packaging (Liu et al., 2021). Peptide-modified vectors can engage alternative cellular receptors, bypassing reliance on canonical receptors such as HSPGs (Liu et al., 2021). Peptide insertion in combination with directed evolution, as well as capsid shuffling has yielded AAV variants with novel tissue tropisms, including those with enhanced EC transduction (Maheshri et al., 2006). In addition to the modified tissue-specificity of capsids, transgene expression specificity can be further restricted by using cell type-specific promoters. For targeting EC, promoters such as ICAM2, FLT1, or eNOS have been used to ensure that even if off-target transduction occurs, the expression of therapeutic gene is restricted to ECs (Liu et al., 2021; Barbon et al., 2023).

**TABLE 1:** AAV capsid multipeptide insertions that enhance endothelial cell tropism.

AAV vector	Peptide sequence	Targeted cells	Species tested	Improvements	Reference
AAVsig	SIGYPLP	Vascular ECs (HUVECs, HSVECs)	*In vitro*	Loss of HSPG recognition, predisposition to less acidic environment in endosomes	Nicklin et al. (2001), Zhang et al. (2019)
AAV2-NDVRAVS	NDVRAVS	HUVECs, human coronary artery endothelial cells	*In vitro*	Susceptible to neutralizing antibodies	Müller et al. (2003), Varadi et al. (2012), Zhang et al. (2019)
AAVmtp	MTPFPTSNEANL	Vena cava	*In vitro,* *in vivo* (mice)	Exploits native tropism	White et al. (2004)
AAV9- NDVRAVS	NDVRAVS	HUVECs, human coronary artery endothelial cells	*In vitro*	Less affected by neutralizing antibodies	Varadi et al. (2012)
AAV-V_EC_	VSSSTPR	HUVECs, iPSC-derived endothelial progenitor cells	*In vitro*	Transduce proliferating and quiescent cells	Zhang et al. (2019)
AAV2-EC71; AAV2-EC73	AEDGVAR; NHPPGGV	Cardiac ECs	*In vitro,* *in vivo* (mice)	Exploits native tropism, Reduced liver transduction and HSPG recognition	Liu et al. (2021)
AAV9mut-B	Undisclosed	HUVECs, murine aortic cells	*In vitro,* *in vivo* (mice)	Non-therapeutic expression, off-target effects	Barbon et al. (2023)
AAV-PSS	DSPAHPS	Brain microvasculature of WT mice	*In vivo* (mice)	Targets healthy ECs	Chen et al. (2009)
AAV-PFG	WPFYGTP	Brain microvasculature of LSD MPSVII	*In vivo* (mice)	Targets diseased ECs	Chen et al. (2009), Chen et al. (2012)
AAV-PHP.eB	TLAVPFK	Brain microvasculature	*In vivo* (mice)	Can cross BBB, off-target effects	Deverman et al. (2016)
AAV2-BR1	NRGTEWD	Microvasculature of brain, spinal cord, retina	*In vitro,* *in vivo* (mice)	Low neuron off-target transduction	Körbelin et al. (2016)
AAV-PHP.V1	TALKPFL	Brain vascular cells, astrocytes, neurons	*In vitro,* *in vivo* (mice)	Can induce immune response, neuron off-target transduction	Ravindra Kumar et al. (2020)
AAV-BI30	NNSTRGG	Arterial, capillary and venous endothelial cells of brain, spinal cord and retina	*In vitro,* *in vivo* (mice, rats)	Minimal neuron off-target transduction	Krolak et al. (2022)
AAV-X1	GNNTRSV	Brain microvasculature	*In vitro,* *in vivo* (mice, rats, marmosets, rhesus macaques), human brain tissue	High-specificity labeling of BECs in rodents, but not primates	Chen et al. (2023)

The first attempts to insert EC-recognizing peptides involved incorporation of the SIGYPLP heptapeptide into position I-587 of the AAV2 capsid (AAVsig), which improved vascular EC transduction. This modification also reduced gene delivery to off-target cells such as hepatocytes and vascular smooth muscle cells. AAVsig cell entry was independent of HSPG binding, the primary receptor required for AAV2 attachment and infection (Nicklin et al., 2001). HSPGs are abundant in the vascular extracellular matrix, where they can trap AAVs and reduce productive transduction of ECs (Pajusola et al., 2002). Therefore, overcoming this limitation enabled efficient endothelial transduction despite the liver and muscle tropism of AAV2, marking an important breakthrough in the development of novel tools to target ECs. In addition, AAVsig exhibited enhanced intracellular trafficking, as evidenced by increased transduction in the presence of bafilomycin A2, an inhibitor of endosomal acidification, although the detailed mechanism remains to be elucidated (Nicklin et al., 2001).

Subsequent work extended these principles to other serotypes. This was because AAV2 is highly susceptible to recognition by immunoglobulin G in human serum, whereas serotypes such as AAV9 or AAV5 are comparatively less affected by neutralizing factors (Boutin et al., 2010; Varadi et al., 2012). An EC-specific 7-mer peptide (NDVRAVS), originally identified by *in vitro* selection from an AAV2 peptide display library (Müller et al., 2003), was introduced at site A589 of the AAV9 capsid. Both AAV2-NDVRAVS and AAV9-NDVRAVS exhibited improved transduction of human coronary artery endothelial cells (HCAECs), with approximately 70% higher efficiency compared with their respective wild type vectors. Additional heptapeptides (SLRSPPS; RGDLRVS) identified from AAV2 or AAV9 libraries yielded even greater enhancements in HCAEC transduction (Varadi et al., 2012).

More recent work refined this approach by generating panels of AAV9 variants containing endothelial-targeting heptapeptides, among which AAV9mut-B demonstrated superior transduction in human umbilical vein endothelial cells (HUVECs) *in vitro* and in murine aortic endothelium *in vivo*. AAV9mut-B achieved higher vector genome copy numbers and stronger transgene expression in aortic tissue compared to wild-type AAV9, although residual off-target expression in the liver and lung reflected incomplete detargeting from AAV9 native tropism. Transgene expression was detected in ECs but remained below therapeutic levels, which underscored the need for further improvements and modifications in the expression cassette (Barbon et al., 2023).

Among peptide-engineered vectors, AAV-VEC (VSSSTPR) represented a great step forward in endothelial targeting. This vector outperformed parent AAV2 as well as first-generation targeting constructs such as AAV-NDVRAVS and AAV2-SIG, achieving a 4- and 13-fold increase in transduction efficiency in HUVECs, respectively (Zhang et al., 2019). Importantly, AAV-VEC efficiently transduced both proliferating and quiescent ECs, a key advantage given that the majority of ECs lining the inner layer of the vessels are non-proliferating. In addition, AAV-VEC demonstrated high potency in modifying induced pluripotent stem cells (iPSC)-derived endothelial progenitor cells (EPC), which home to damaged tissue or growing vessels. Such ECs modified *ex vivo* were more susceptible toward AAV-VEC transduction, reaching almost 100% transduction efficiency with genome-of-infection (GOI) of only 1,000 (Zhang et al., 2019).

Other engineered variants further illustrate the versatility of peptide insertion. The vectors AAV2-AEDGVAR (EC71) and AAV2-NHPPGGV (EC73) showed enhanced cardiac EC transduction with reduced uptake by liver ECs. Both vectors exhibited reduced heparin binding, but retained dependence on AAVR as the primary internalization receptor (Liu et al., 2021). Similarly, the venous EC-targeted AAVmtp, containing the MTPFPTSNEANL peptide, accumulated preferentially in the vena cava, while minimizing liver uptake and exhibited delayed blood clearance. Competitive inhibition with heparin or free peptides confirmed that uptake was predominantly peptide mediated rather than reliant on native AAV tropism. Nonetheless, original vector tropism can still be exploited to reach ECs in certain tissues, such as the liver and cardiac or skeletal muscle endothelium (White et al., 2004).

Mechanistically, peptide modifications influence both receptor engagement and intracellular trafficking. In several cases, modified vectors such as AAVsig (Nicklin et al., 2001) and AAVmtp (White et al., 2004) demonstrated improved gene transfer efficiency in the presence of heparin, which competes with HSPGs for capsid binding and prevents ECM sequestration, thereby allowing the peptide-mediated entry route to predominate. These findings suggest that AAV capsid engineering can overcome both extracellular and intracellular barriers to EC transduction by altering virus-host cell interactions at multiple levels.

Targeting brain microvasculature has emerged as a particularly compelling, yet challenging objective in gene therapy. Endothelial dysfunction contributes to the progression of neurological disorders, as blood-brain barrier (BBB) impairment can accelerate neurodegeneration and neuroinflammation (Körbelin et al., 2016). Therefore, efficient and specific gene delivery to brain ECs remains a critical challenge for gene therapy. Similarly to strategies used for peripheral EC targeting, several capsid-modified AAVs have been engineered to selectively transduce cerebrovascular ECs (Table 1). A common approach again involves inserting tissue-specific heptapeptides at amino acid site 588–589 to redirect vector tropism toward the vasculature of the central nervous system (CNS) (Chen et al., 2009; Chen et al., 2023; Deverman et al., 2016; Körbelin et al., 2016; Krolak et al., 2022).

Using CREATE (Cre-recombination-based AAV targeted evolution) and its multiplex version, developed to enhance AAV transcytosis across the BBB and subsequent CNS transduction, several cell-specific peptides have been identified, including AAV-PHP.eB (TLAVPFK) (Deverman et al., 2016) and AAV-PHP.V1 (TALKPFL) (Ravindra Kumar et al., 2020), which show enhanced tropism for brain ECs in addition to the main target CNS. These vectors transduce the CNS with an efficiency ∼40 times and ∼120 times higher, respectively, compared to wild-type AAV9, due to the ability to traverse the BBB. They recognize the specific receptor - Ly6A (also known as Sca-1), a surface protein expressed on ECs, which is essential for capsid transcytosis across the BBB (Grimm and Nonnenmacher, 2023).

Subsequent engineering efforts focused on utilizing this approach and improving the specificity for cerebrovascular ECs instead of neurons. AAV-BR1 (NRGTEWD) showed strong and stable transgene expression in the CNS with minimal or no off-target activity in other organs. Compared to wild-type AAV2, AAV-BR1 achieved ∼650-fold higher brain expression. Transduction was observed in retina, spinal cord and brain microvascular cells *in vivo* in mice, as well as immortalized human cerebral microvascular ECs (hCMEC/D3) *in vitro*, predominantly in capillaries, with markedly reduced efficiency in larger vessels. Neuronal transduction was also much lower than with AAV-PHP.eB or AAV-PHP.V1, likely reflecting the use of different surface receptors. Unlike the variant AAV-PHP.eB, which depends on Ly6A, the cell entry of AAV-BR1 is mediated by the type I transmembrane protein KIAA0319L (AAVR). Its therapeutic potential was demonstrated by AAV-BR1-mediated delivery of the NEMO gene into a mouse model of incontinentia pigmenti, which saved the survival of brain ECs (Körbelin et al., 2016).

AAV-BI30 (NNSTRGG) improved upon AAV-BR1 by efficiently transducing the arterial, capillary, and venous endothelium in the brain, spinal cord, and retina of mice and rats *in vivo*, as well as in hCMEC/D3 *in vitro*. AAV-BI30 exhibited twice the specificity for large vessel ECs and 1.5-fold higher spinal cord transduction compared to AAV-BR1. Similar to AAV-BR1, its cell entry is Ly6A-independent, and neuronal off-target transduction is minimal. Neuronal off-targeting decreased in the order: PHP.V1 > BR1 > BI30. Incorporation of three repeats of the hepatocyte-specific miR-122 target sequence into the 3′UTR of the construct further enabled transduction of the lung, aorta, and kidney vasculature. Finally, AAV-BI30-mediated knockdown of caveolin-1 confirmed brain EC-specific functional targeting (Krolak et al., 2022).

Recent research led to the development of AAV-X1 (GNNTRSV), which efficiently transduces cerebrovascular ECs not only in rodents (mice and rats) with high specificity, but also in non-human primates such as marmosets and rhesus macaques, as well as in *ex vivo* human brain slices. Expression levels were sufficient to restore lost phenotypes in Sparcl1/Hevin knockout mice. A major advantage of this vector is that the targeting peptide can be incorporated into multiple AAV backbones (e.g., AA9 and AAV1), enabling repeated systemic administration without inducing neutralizing immune responses (Chen et al., 2023).

Distinct endothelial phenotypes have also been exploited in health versus disease. AAV-PFG (WPFYGTP) was engineered to transduce the cerebral vasculature of mice with mucopolisaccharidosis type VII (MPS VII), a lysosomal storage disease (LSD), whereas AAV-PPS (DSPAHPS) preferentially targets healthy cells. AAV-PFG binding to brain ECs is mediated by interaction with glycosaminoglycans (GAGs), particularly chondroitin sulfate, which accumulates in MPS VII as a result of defective lysosomal degradation. The systemic delivery of AAV-PFG encoding lysosomal enzymes restored substrate degradation, reduced lysosomal storage, reversed cognitive deficits in mice, and improved survival. Peptide insertion compromised HSPG binding but did not affect viral viability. Liver transduction was preserved, while brain uptake was significantly enhanced (Chen et al., 2009; Chen et al., 2012).

Endothelial heterogeneity has driven the development of diverse AAV capsid–engineering strategies that yield vectors with distinct but complementary outcomes. Peptide insertion into AAV capsids has enabled improved endothelial transduction by altering receptor usage, reducing off-target tropism, and enhancing intracellular trafficking. Different engineered vectors achieve varying degrees of specificity, efficiency, and tissue coverage - ranging from peripheral and cardiac endothelium to highly specialized brain microvascular targets. In the CNS, successive generations of vectors show complementary strengths: some maximize BBB crossing and CNS access, others prioritize endothelial specificity, reduced neuronal off-targeting, disease-state selectivity, or cross-species translatability (Table 1). Collectively, these outcomes indicate that no single vector is universally optimal; instead, the expanding toolkit of AAV variants provides complementary solutions tailored to specific vascular beds, disease contexts, and therapeutic goals.

## Conjugating antibodies to AAV capsid

Chemical capsid modification enables precise endothelial retargeting without the need for genetic engineering. A notable example is the conjugation of a single-chain antibody (scFv) against vascular cell adhesion molecule-1 (VCAM-1), an inflammation-induced endothelial marker, to recombinant AAV6. Using sortase A-mediated site-specific coupling, the scFv was functionalized with an orthogonal click chemistry group to preserve antigen-binding capacity. AAV6 capsids were modified with 4-azidophenyl glyoxal (APGO), which forms covalent adducts at arginine residues in the heparin-binding region, thereby abrogating native tropism. The two components were subsequently linked via strain-promoted azide-alkyne cycloaddition, producing an antibody-decorated AAV6 with significantly reduced off-target transduction of hepatic cells and a four-to five-fold increase in EC transduction *in vitro*. Although *in vivo* data were not reported, these findings highlight antibody conjugation as a promising chemical strategy for endothelial-specific AAV delivery (Pearce et al., 2019).

## Polymer coating enhancing EC specificity

Surface modification of AAV vectors with synthetic polymers has emerged as an innovative, non-genetic approach to retarget transduction toward ECs. Polymer coatings can modulate vector tropism, enhance circulation stability, and shield viral capsids from host immune recognition (Kaygisiz and Synatschke, 2020).

PEG is widely used to sterically shield AAV capsids from immune detection, thereby reducing opsonization and neutralization by pre-existing antibodies. PEGylation can also prolong vector circulation time, increasing the likelihood of target tissue engagement (Lee et al., 2005). Polyethylene imine (PEI), a highly cationic polymer, enhances cellular uptake through electrostatic interactions with negatively charged membranes and facilitates endosomal escape through its “proton sponge” effect. In acidic endosomal compartments, PEI buffers pH and induces osmotic swelling, leading to endosomal rupture before lysosomal degradation occurs. However, the utility of PEI is limited by dose-dependent cytotoxicity, which has spurred the development of safer derivatives and optimized dosing strategies (Akinc et al., 2005; Kaygisiz and Synatschke, 2020).

Poloxamers, thermoresponsive triblock copolymers of poly(ethylene oxide)–poly(propylene oxide)–poly(ethylene oxide) (PEO-PPO-PEO), form micellar structures capable of encapsulating viral particles. This micellar incorporation has been reported to improve vector stability and transduction efficiency, possibly by protecting capsids from enzymatic degradation and facilitating membrane fusion (Feldman et al., 1997; Kaygisiz and Synatschke, 2020). Lastly, dendrimers, monodisperse and highly branched macromolecules with a high density of terminal functional groups, internal cavities, and precise molecular geometry, offer another versatile scaffold. These features make them ideal scaffolds for attaching targeting ligands, shielding capsids, and carrying additional cargo. Dendrimers can be conjugated with peptides, antibodies, or polymers, enabling modular assembly of multifunctional AAV delivery platforms (Abbasi et al., 2014). Beyond these, other classes of nanomaterials – including polysaccharides (Sailaja et al., 2002), lipids (Malaekeh-Nikouei et al., 2018), hydrogels (Shin and Shea, 2010; Seidlits et al., 2013), graphene (Kaygisiz and Synatschke, 2020), silica (Kangasniemi et al., 2009), magnetics nanoparticles (Scherer et al., 2002), and small molecules (Nicolson et al., 2016) – are also being investigated for their ability to modify viral tropism and delivery kinetics (Kaygisiz and Synatschke, 2020).

A notable example of polymer-assisted endothelial retargeting involves the use of second-generation amine-terminated polyamidoamine (G2-PAMAM) dendrimers as scaffolds for attaching endothelial-affine peptides (EPs) to the AAV capsid. In this system, dendrimers are first PEGylated using OPSS-PEG-NHS (OPN) linkers, which create a stable matrix for EP conjugation, yielding PEG-G2-EP-AAV complexes. This strategy enables non-genetic surface modification of AAV capsids, significantly enhancing EC targeting while preserving capsid integrity. An illustrative case is the coating of AAV9-Cre capsids with G2-PAMAM dendrimers linked to CNN peptide (CNNSGMRN), an EC-targeting ligand identified by phage display biopanning. The resulting G2-CNN-AAV9-Cre vector demonstrated markedly improved transduction of ECs in multiple vascular beds, including the aorta, liver, diaphragm, spleen, and brain microvasculature (Bozoglu et al., 2022).

The positive surface charge conferred by the dendrimer coating enhanced cellular uptake, while the CNN motif mediated receptor-specific internalization. This process likely occurs via stabilin-2 (STAB2), a phosphatidylserine receptor expressed in liver and bone marrow sinusoidal endothelium (Park and Kim, 2019). Interestingly, although native AAV9 tropism is myotropic – particularly towards skeletal muscle and heart – this was leveraged rather than eliminated in the retargeting strategy. Coating AAV9 with G2-CNN preserved the natural biodistribution of the serotype, but redirected expression to vascular cells (Bozoglu et al., 2022).

The functional efficacy of the G2-CNN-AAV9 vectors has been demonstrated in multiple disease models. In a blood pressure regulation model, AAV9-mediated delivery of gRNA targeting eNOS led to significant phenotypic changes, including increased blood pressure resulting from reduced nitric oxide production in ECs. In an atherosclerosis model, transduction of vascular ECs with S1FG, a leukocyte adhesion inhibitor, reduced the recruitment of inflammatory cells (Bozoglu et al., 2022). In a Marfan syndrome model, overexpression of regnase-1 in aortic tissues attenuated inflammation, prevented elastin degradation, and inhibited aortic aneurysm formation (Noormalal et al., 2024). Together, these studies demonstrate that G2-CNN-AAV9 can efficiently deliver genetic cargo to ECs, achieving stable transgene expression with substantial therapeutic effects across diverse vascular disease models (Bozoglu et al., 2022).

Coating AAV capsids with PEGylated dendrimers and endothelial-affine peptides offers multiple advantages. The system is highly modular, as different peptides can be attached to retarget the vector toward specific EC subtypes. Native tropism can be preserved, allowing vectors to accumulate in their usual tissues while endothelial expression can be fine-tuned through EC-specific promoters and peptides. This feature can be exploited, for example, to transduce the muscle endothelium. In contrast, complete detargeting from the natural tropism of a serotype requires genetic capsid engineering, which often compromises vector stability or transduction efficiency. Polymer coating preserves capsid integrity, avoids folding issues, and circumvents potential immune recognition triggered by altered capsid sequence, as any surface modifications are non-genetic (Bozoglu et al., 2022; Noormalal et al., 2024). A particularly promising avenue in mice would be to apply this coating strategy to AAV4, which has intrinsic endothelial tropism, especially in the lung. Combining AAV4 with G2-EPs can enable highly specific delivery to distinct vascular beds, without extensive off-target transduction.

## Discussion

The intrinsic resistance of ECs to AAV transduction remains a major obstacle in the development of vascular-targeted gene therapies. Although the endothelium constitutes the first cellular interface encountered after systemic administration, most natural AAV serotypes display negligible affinity for ECs compared with hepatocytes, myocytes or neurons, toward which they show strong natural tropism (White et al., 2004; Wang et al., 2024). This restricted tropism has hindered progress in the application of AAVs for diseases in which endothelial dysfunction plays a central role. Among the naturally occurring serotypes, AAV4 has recently been recognized to possess a relatively higher affinity for ECs, particularly within the murine pulmonary vasculature. Its unique ability to bind O-linked sialic acids and enter cells via the AAVR-independent pathway distinguishes it from other serotypes (Walkey et al., 2025). However, this endothelial tropism appears to be species-specific. Current evidence indicates that AAV4 fails to efficiently transduce ECs derived from rats or humans, instead showing preferential uptake by hepatocyte-like cell lines (Chen et al., 2005; Stamataki et al., 2025). The available data remain limited and inconsistent, highlighting the need for further comparative studies.

Given the poor endothelial transduction of native serotypes, genetic capsid engineering has become a central strategy for redirecting AAV tropism. Transduction efficiency can be markedly improved by modifying receptor binding profiles, detargeting AAVs from HSPGs abundant in the endothelial extracellular matrix and enhancing resistance to endosomal degradation (Nicklin and Baker, 2002; Pajusola et al., 2002). Insertion of short EC-specific peptides into the capsid sequence has enabled selective targeting of distinct vascular beds, including those of the brain, kidney and heart, often accompanied by reduced hepatic uptake (Nicklin et al., 2001; White et al., 2004; Liu et al., 2021; Barbon et al., 2023). Recent studies have focused particularly on the targeting of the cerebrovascular endothelium and the development of variants capable of transcytosis across the BBB (Chen et al., 2009; Deverman et al., 2016; Ravindra Kumar et al., 2020; Krolak et al., 2022; Chen et al., 2023). These findings hold promise for the treatment of neurological and neurovascular disorders, although targeting larger systemic vessels remains comparatively underexplored.

Non-genetic approaches to endothelial retargeting have also shown considerable potential. The conjugation of capsids with antibodies directed against EC-specific markers (Pearce et al., 2019) or the coating with polymers carrying endothelial-affine peptides (Bozoglu et al., 2022; Noormalal et al., 2024) can further enhance vascular specificity. For instance, the PEGylated dendrimer coating provides modular control over tropism while protecting the vector from neutralizing antibodies and prolonging circulation time (Kaygisiz and Synatschke, 2020).

The most promising strategy for endothelium-targeted gene therapy is the use of in vivo-evolved endothelial-tropic AAV capsids. These vectors are expected to exhibit preferential transduction of ECs over hepatocytes, function effectively following systemic administration, and possess strong physiological relevance, as they are selected under conditions that reflect blood flow, shear stress and immune pressure. Additional improvements in vector specificity can be achieved through the use endothelial-specific promoters or miRNA-based detargeting strategies. Future endothelial-targeted vectors will likely integrate multiple strategies, combining peptide-based capsid engineering with chemical and polymer-based surface modifications to achieve efficient, selective and species-translatable endothelial transduction. Such multifaceted systems may unify precise receptor recognition, reduced immunogenicity and enhanced intracellular trafficking, ultimately enabling safe and effective delivery of therapeutic genes to the endothelium across diverse organs and species, bringing AAV-based vascular therapies closer to clinical application.
